# Lentivirus-mediated silencing of the *PTC1* and *PTC2* genes promotes recovery from spinal cord injury by activating the Hedgehog signaling pathway in a rat model

**DOI:** 10.1038/emm.2017.220

**Published:** 2017-12-15

**Authors:** Ya-Dong Zhang, Zhong-Sheng Zhu, Dong Zhang, Zhen Zhang, Bin Ma, Shi-Chang Zhao, Feng Xue

**Affiliations:** 1 Department of Orthopaedics, Shanghai Fengxian Central Hospital, South Campus of the Sixth People’s Hospital Affiliated to Shanghai Jiao Tong University, Shanghai, People’s Republic of China; 2 Department of Spine Surgery, Shanghai East Hospital, School of Medicine, Tongji University, Shanghai, People’s Republic of China; 3 Department of Orthopedics, the Affiliated Sixth People’s Hospital of Shanghai Jiao Tong University, Shanghai, People’s Republic of China

## Abstract

This study aimed to investigate the effect of Patched-1 (*PTC1*) and *PTC2* silencing in a rat model, on Hedgehog (Hh) pathway-mediated recovery from spinal cord injury (SCI). An analytical emphasis on the relationship between the sonic hedgehog (Shh) pathway and nerve regeneration was explored. A total of 126 rats were divided into normal, sham, SCI, negative control (NC), PTC1-RNAi, PTC2-RNAi and PTC1/PTC2-RNAi groups. The Basso, Beattie and Bresnahan (BBB) scale was employed to assess hind limb motor function. Quantitative real-time polymerase chain reaction and western blotting were performed to examine the mRNA and protein levels of PTC1, PTC2, Shh, glioma-associated oncogene homolog 1 (Gli-1), Smo and Nestin. Tissue morphology was analyzed using immunohistochemistry, and immunofluorescent staining was conducted to detect neurofilament protein 200 (NF-200) and glial fibrillary acidic protein (GFAP). The PTC1/PTC2-RNAi group displayed higher BBB scores than the SCI and NC groups. Shh, Gli-1, Smo and Nestin expression levels were elevated in the PTC1/PTC2-RNAi group. PTC1 and PTC2 mRNA and protein expression was lower in the PTC1/PTC2-RNAi group than in the normal, sham and SCI groups. Among the seven groups, the PTC1/PTC2-RNAi group had the largest positive area of NF-200 staining, whereas the SCI group exhibited a larger GFAP-positive area than both the normal and the sham groups. The Shh pathway may provide new insights into therapeutic indications and regenerative recovery tools for the treatment of SCI. Activation of the Hh signaling pathway by silencing *PTC1* and *PTC2* may reduce inflammation and may ultimately promote SCI recovery.

## Introduction

Spinal cord injury (SCI) is a devastating medical condition characterized by motor, cardiac, bowel, sensory and bladder dysfunction.^1^ SCI is understood to progress in two stages: first through the mechanical disruption of various nervous tissues, followed by a serious perturbation of the local blood supply manifested by the secretion of pro-inflammatory mediators and neurotoxins from both resident and invading cells.^2^ Worldwide, more than 30 000 new SCI cases occur each year, exhibiting relatively high morbidity, which requires long-term physical and medical care for SCI patients.^3^ A range of approaches have been adopted for treating SCI, and unfortunately, all have achieved only relatively limited success.^4^ Using various degenerative mechanisms, SCI not only gives rise to acute and progressive secondary disruption of local and distant nervous tissues, but it also causes a series of endogenous regenerative and neuro-protective reactions.^5^ A comprehensive investigation of such mechanisms may facilitate current and future SCI treatment approaches. It has been suggested that the Sonic hedgehog (Shh) signaling pathway may play a vital role in nerve regeneration after injury. Previous research has provided evidence alluding to the notion that nerve function is improved by Shh treatment in diabetic models of neuropathy.^6, 7^ Thus, it has been hypothesized that the Shh signaling pathway may contribute to nerve regeneration after SCI and may promote the recovery of SCI patients.

The hedgehog (Hh) signaling pathway is crucial for the patterning and growth of various tissues during embryonic development.^8^ Both Desert (Dhh) and Indian hedgehog (Ihh) belong to the Hh family and are well-described ligands in this signaling pathway.^9^ Shh, a key protein involved in craniofacial morphogenesis, promotes cell proliferation during embryogenesis in a tissue-specific manner by regulating epithelial–mesenchymal interactions.^10^ The Shh signaling pathway initiates the destruction of a large cytoplasmic protein complex and promotes the release of oncogene homolog (Gli) transcription factors. The Shh-Gli signaling pathway plays diverse roles in mammalian development, including the regulation of cell fate determination, cell proliferation, cell differentiation, and cell survival and patterning of the central nervous system.^11^ The Hh proteins secreted in the Hh signaling pathway interact with the membrane-bound receptors Patched-1 (PTC1) and Patched-2 (PTC2), relieving the patched-mediated repression of the signal transducer smoothened (Smo) and activating glioma-associated transcription factors (Gli-1, Gli-2 and Gli-3) and promoting the transcription of downstream genes such as *Ptc1*, *Ptc2* and *Gli1*.^12^ Considering that PTC1 negatively regulates Hh signaling during embryonic development,^13^ the present study aimed to investigate whether *PTC1* and *PTC2* silencing could promote SCI recovery via the Hh signaling pathway.

## Materials and methods

### Ethics statement

The experiment was conducted with approval from the Ethics Committee of Shanghai Fengxian Central Hospital, Branch of the Sixth People’s Hospital Affiliated to Shanghai Jiao Tong University. All components of the study were completed in strict accordance with all relevant regulations.

### Experimental animals

A total of 126 specific-pathogen-free (SPF) male Wistar rats were selected for the purposes of the study (weight: 200–220 g; age: 7 weeks old; license number: SCXK Sichuan Province 2016; Beijing Huafukang Biotechnology, Beijing, China). The chosen rats were fed according to the standard procedures.

### Establishment of the SCI rat model

The abdominal cavity of the rats was injected with 10% chloral hydrate anesthetic. Following identification of the spinal cord region (T7-T9 segment), a median incision (3–4 cm) was created, followed by an incision layer-by-layer incisions through the skin, subcutaneous tissues and fascia to separate the lateral muscular tissues and to expose the neural plates. A micro-rongeur (Friedman-Person, 14 cm-diameter, Fine Science Tools, Hunan Yuanxiang Bio-tech, Changsha, Hunan, China) was used to fracture the spinous process and neural plate to fully expose the spinal cord. After cord exposure, SCI rat models were established using a modified Allen’s method. A stainless steel bar (diameter: 2.5 mm; weight: 5.0 g) was vertically released along a graduated tube from a height of 5 cm and bumped with a plastic bar (diameter: 3 mm) at the bottom to damage the rat spinal cords, which were immediately weighed and sutured using 0 stitches. Successful establishment of the rat model was based on whether the spinal cord exhibited hemorrhage and edema, the lower limbs of the rats fluttered contractively, their tails swayed spastically and finally flaccid paralysis was manifested.^14^ After successful establishment of the SCI rat models, hemostasis by compression was performed using a gelatin sponge until no obvious bleeding was observed at the operative site. When the spinal cord was clearly exposed, the rats were fixed on an intravenous stand.

### Lentivirus-mediated RNAi targeting of *PTC1* and *PTC2*

Short-hairpin RNAs (shRNAs) targeting *PTC1* and *PTC2* were designed based on the *PTC1* (no. AB000848.1) and *PTC2* (no. AB000846.1) sequences from GenBank. Two siRNAs, siRNA-1-PTC1 (positive-sense strand: 5′-acagttcgaccctttggaa-3′ negative-sense strand: 5′-ttccaaagggtcgaactgt-3′) and siRNA-2-PTC1 (positive-sense strand: 5′-gaatcccacagatcccgaa-3′ negative-sense strand: 5′-ttcgggatctgtgggattc-3′) were designed to target sequences starting at *PTC1* nucleotides 121 and 181, respectively. Similarly, two siRNAs, siRNA-1-*PTC2* (positive-sense strand: 5′-cctaaatccaatgcagctg-3′ negative-sense strand: 5′-cagctgcattggatttagg-3′) and siRNA-2-*PTC2* (positive-sense strand: 5′-aagagacgggttcccaaca-3′ negative-sense strand: 5′-tgttgggaacccgtctctt-3′) were designed to target sequences starting at *PTC2* nucleotides 61 and 161, respectively. The DNA oligonucleotides were synthesized using Sangon Biotech (Shanghai, China). The positive and negative single-stranded DNAs were annealed and recombined with the RetroQ plasmid digested with BamHI and EcoRI to form the ReteoQ-shRNA plasmids. PCR-amplified sequences from *PTC1* (forward: 5′-tgctaaactacaaactggaa-3′ reverse: 5′-gctcattgattggaatttcc-3′) and *PTC2* (forward: 5′-gaccctccttaacgaattgt-3′ reverse: 5′-gctccttgctgggcatctct-3′) were recombined with BamHI and EcoRI digested at pEGFP-N1 to construct the pEGFP-PTC plasmids. The ReteoQ-shRNA and pEGFP-PTC plasmids were transformed, filtrated, amplified and co-transfected. Third-generation lentiviral particles containing effective siRNAs were generated using the ReteoQ-shRNA packaging plasmid transfected with polyethyleneimine and stored as frozen stocks. Viral titers were determined using serial dilutions. Plasmids were screened for silencing via co-transfection with the GFP reporters. Seventy-two hours after co-transfection of ReteoQ-shRNA and pEGFP-PTC plasmids, fluorescence generated from the ReteoQ-shRNA plasmids was observed. The establishment of ReteoQ-shRNA plasmids was deemed successful if no green fluorescence was observed. Rat dorsal root ganglion (DRG) cells were cultured for 5 days, after which the cells were infected with lentivirus, cultured for another 48 h and subsequently lysed. Quantitative real-time polymerase chain reaction (qRT-PCR) and western blotting were performed to detect the expression of PTC mRNAs and proteins to evaluate viral interference.

### Experimental groups and viral intervention

SCI rat models were assigned to the following seven groups: (1) normal group (healthy rats); (2) sham group (rats that had undergone anesthetization, neural plate fracture and exposure of the spinal cord without any injury treatment); (3) SCI group; (4) negative control (NC) group (SCI rats that received a spinal cord injection of 8 μl lentivirus (10^7^ TU ml^−1^) containing ineffective shRNA); (5) *PTC1*-RNAi group (SCI rats that received a spinal cord injection of 8 μl of lentivirus (10^7^ TU ml^−1^) with *PTC1*-shRNA); (6) *PTC2*-RNAi group (SCI rats that received a spinal cord injection of 8 μl of lentivirus (10^7^ TU ml^−1^) with *PTC2*-shRNA) and (7) *PTC1/PTC2*-RNAi group (SCI rats that received a spinal cord injection of 8 μl of lentivirus (10^7^ TU ml^−1^) with *PTC1/PTC2*-shRNA). After the models were successfully established, the rats underwent thorough hemostasis and suturing of their respective incisions. Within 1–3 days after the operation, rats in the NC group were injected with antibiotics to help fight infection. Rat bladders were pressed gently two to three times daily until voluntary urination was obtained.

### Evaluation of hind limb motor function

The hind limb motor function of the rats was evaluated using the Basso, Beattie and Bresnahan (BBB) scale on the 1st, 7th, 14th, 21st and 28th day after SCI.^15^ Rats were placed in a 125 cm × 125 cm open space and were allowed to move freely. Hind limb motor function and body control in addition to all other relevant indicators were evaluated via attraction to stimuli for 4 min per evaluation. The 21-point BBB scale was used to grade the hind limb motor function of rats. In general, 0 points indicate complete paralysis and 21 points indicate normal locomotion. Scores ranging between 1 and 20 indicate a corresponding level of performance of the rat hind limbs. The detailed and basic scoring parameters were in accordance with a previous study,^16^ and a three-part evaluation was included. First, the degree of agility and other details relevant to the hind limb joint movements were evaluated; second, gait and coordination were assessed; third, fine movement of the rat toes was tallied.

### qRT-PCR

After the evaluation of hind limb motor function, rat spinal cords were collected and digested followed by cell lysis. Total RNA was extracted using an RNA kit (Omega Bio-tek, Norcross, GA, USA). Reverse-transcription was conducted in accordance with the instructions provided with the reverse transcription kit (Thermo Fisher Scientific, Sunnyvale, CA, USA). The cDNA was used as a template for PCR. Primer 5.0 software was employed to design primers, and β-actin was used as an internal reference. The primer sequences were as follows: Shh: forward: 5′-ccgaacgatttaaggaactcac-3′, reverse: 5′-tgtctttgcacctctgagtcat-3′ Gli-1: forward: 5′-ctctgctgactctgggatatg-3′, reverse: 5′-gatcaggataggagcctgctg-3′ PTC1: forward: 5′-tgctaaactacaaactggaa-3′, reverse: 5′-gctcattgattggaatttcc-3′ PTC2: forward: 5′-gaccctccttaacgaattgt-3′, reverse: 5′-gctccttgctgggcatctct-3′ Nestin: forward: GCGGGGCGGTGCGTGACTAC, reverse: AGGCAAGGGGGAAGAGAAGGATGT; Smo: forward: GCAGTTCCTCGGCTGCCTC, reverse: AGCCTCCATTAGGTTAGTGCG; and β-actin: forward: 5′-tgggacgacatggagaaaa-3′, reverse: 5′-ctggaaggtggacagcgag-3′. The threshold cycle (Ct) values were determined and used to quantify the relative levels of PTC1, PTC2, Gli-1 and Shh mRNAs using the 2^−ΔΔCt^ method.

### Western blotting

The collected 100 mg of spinal cord tissue from rats in all 7 groups was washed with phosphate-buffered saline (PBS). The total protein amount was extracted according to the procedural instructions provided by the tissue protein extraction kit (No. P060091; Shanghai Andi Biotechnology Co., Ltd, Shanghai, China) and separated by sodium dodecyl sulfate polyacrylamide gel electrophoresis (SDS-PAGE). Next, the total protein was electrically transferred onto a polyvinylidene fluoride (PVDF) membrane for detection. Antibodies were used at the following dilution ratios: rat anti-PTC1 (Santa Cruz; SC-6147; 1: 1000), rat anti-PTC2 (Proteintech; 55091-1-AP; 1: 1000), rat anti-Shh (Santa Cruz; SC-365112; 1: 1000), rat anti-Gli-1 (Santa Cruz; SC-515751; 1: 1000) and rat anti-β-actin (Santa Cruz; SC-7210; 1: 800) served as an internal reference. Membranes were incubated overnight at 4 °C and then added to horseradish peroxidase (HRP)-marked secondary antibodies (1: 200; #3999; cell signaling) for 2 h at room temperature. The gel imaging system (Gel Doc XR+, Bio-Rad Laboratories, Inc., Hercules, CA, USA) was used to obtain photographs, and ImageJ software was used to test gray levels.

### Hematoxylin and eosin staining

On the 28th day following motor function evaluation, the rats were killed, and spinal cord tissues were dissected and processed for preparation of paraffin-embedded sections. The tissues were fixed in 4% methyl alcohol solution, paraffin-embedded, sectioned and dewaxed. Sections were washed with PBS and stained with hematoxylin and eosin (HE) as follows. First, sections were fixed with acetone for 2 min, washed with water for 1–2 s, and then stained with hematoxylin for 5 min at room temperature. The sections were then washed with water for 5–10 min, immersed in 1% acidic alcohol for 5–10 s and rinsed twice (2 min per rinse) with distilled water. Eosin solution was added to re-stain the sections for 1–2 min. The sections were then washed with distilled water for 1–2 s and dehydrated using a gradient ethanol series (80%, 90% and 100% 30 s per step). Finally, the sections were immersed in xylene I (5 min) and xylene II (5 min) and sealed with neutral gum. Stained tissues were observed under an optical microscope.

### Immunohistochemistry

On the 28th day after motor function evaluation, the rats were killed, and spinal cord tissues were dissected and processed to prepare paraffin-embedded sections. The tissues were fixed in a 4% methyl alcohol solution, paraffin-embedded, sectioned, dewaxed and washed with PBS. The dewaxed and hydrated sections were placed in boiling buffer (citrate buffer; pH=6.0) for 10 min. Each section was then incubated in one drop of 3% H_2_O_2_ for 10 min and in one drop of primary monoclonal antibodies (Neurofilament-200; NF-200; 1: 500 or glial fibrillary acidic protein; GFAP; 1: 200; Fuzhou Maixin Biotech., Fujian, China) for 2 h. The sections were washed with PBS, incubated with avidin-conjugated secondary antibodies (1: 800; Fuzhou Maixin Biotech.) for 20 min, washed again with PBS and incubated with a drop of HRP-marked rabbit anti-mouse polymer for 30 min. Freshly prepared diaminobenzidine (3,3′-diaminobenzidine, DAB) solution was added to each section (one drop), and the staining process was monitored for 5 min under a microscope. Sections were then counterstained, differentiated and blued. Finally, the sections were dehydrated, dried, sealed, aired and photographed. The criteria for scoring were based on positive astrocytes and neurofilaments (previously described).^17^ When 50 high-power fields were counted, the percentage of positive cells of 1000 cells was used to indicate the level of expression, with <50% denoted as (−) and ⩾50% as (+).

### Statistical analysis

SPSS 19.0 software (SPSS, Chicago, IL, USA) was employed for statistical analysis. Measurement data are displayed as the mean±s.d. Comparisons between two groups were performed using the Independent Sample’s *t*-test, while comparisons among multiple groups were conducted by one-way analysis of variance (ANOVA). Multiple comparisons of means were performed using the least significant difference method when there were no differences in the variances or by the Games–Howell method when there were differences in the variances. In addition, the cell-staining data are presented as percentages/ratios. The Chi-square test was also conducted for comparative analysis. *P*<0.05 indicated statistical significance.

## Results

### Successful construction of siRNA plasmids

**Figure 1:**
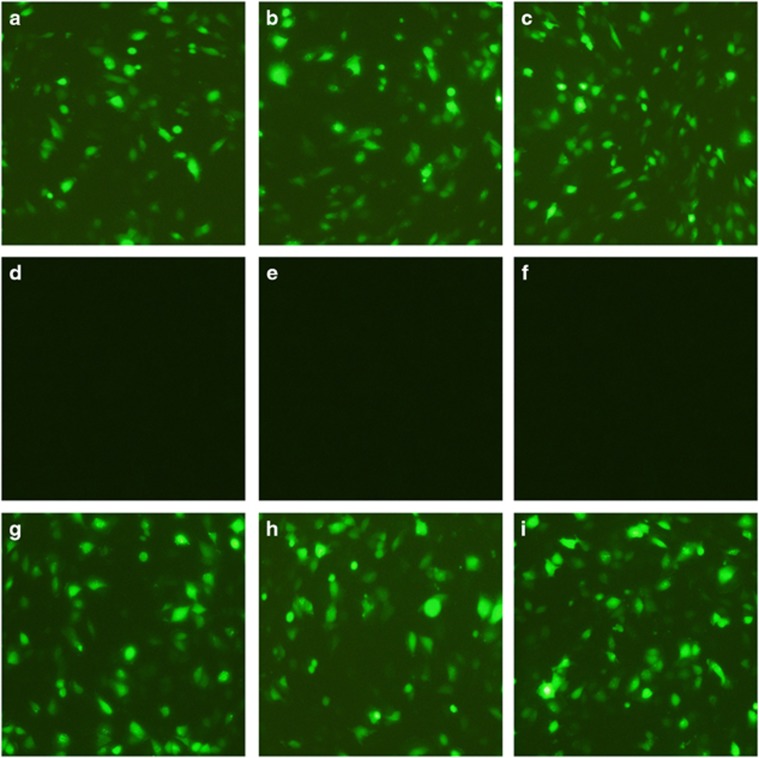
Representations of images of cells transfected with PTC1-RNAi, PTC2-RNAi and PTC1/PTC2-RNAi plasmids using fluorescence microscopy (× 200). Notes: (**a**) cells transfected with the pEGFP-PTC1 plasmid; (**b**) cells transfected with the pEGFP-PTC2 plasmid; (**c**) cells transfected with the pEGFP-PTC1/2 plasmid; (**d**) cells co-transfected with pEGFP-PTC1+ReteoQ-shRNA-1-PTC1 plasmids; (**e**) cells co-transfected with pEGFP-PTC2+ReteoQ-shRNA-1-PTC2 plasmids; (**f**) cells co-transfected with pEGFP-PTC1/2+ReteoQ-shRNA-1-PTC1/2 plasmids; (**g**) cells co-transfected with pEGFP-PTC1+ReteoQ-shRNA-2-PTC1; (**h**) cells co-transfected with pEGFP-PTC2+ReteoQ-shRNA-2-PTC2 plasmids; (**i**) cells co-transfected with pEGFP-PTC1/2+ReteoQ-shRNA-2-PTC1/2 plasmids; EGFP, enhanced green fluorescent protein; PTC1, Patched 1; PTC2, Patched 2.

The fluorescence microscopy results revealed green fluorescence from the pEGFP-PTC1, pEGFP-PTC2 and pEGFP-PTC1/2 plasmids (Figure 1). Seventy-two hours after co-transfection of ReteoQ-shRNA-1-PTC1, ReteoQ-shRNA-1-PTC2 or ReteoQ-shRNA-1-PTC1/2 and pEGFP-PTC, no green fluorescence was observed, which indicated that the PTC-RNAi plasmids had suppressed expression of the complementary pEGFP-PTC reporters. This result indicated the successful construction of the siRNA-1-PTC1, siRNA-1-PTC2 and siRNA-1-PTC1/2 plasmids. By contrast, 72 h after co-transfection of ReteoQ-shRNA-2-PTC1, ReteoQ-shRNA-2-PTC2 or ReteoQ-shRNA-2-PTC1/2 and pEGFP-PTC, green fluorescence was still visible, indicating that the siRNA-2-PTC1, siRNA-2-PTC2 and siRNA-2-PTC1/2 plasmids had not been successfully constructed. Lentiviral particles in the third generation containing effective ReteoQ-shRNA-1-PTC1, ReteoQ-shRNA-1-PTC2 and ReteoQ-shRNA-1-PTC1/2 plasmids were obtained using the polyethyleneimine method and were used for subsequent experiments.

### mRNA and protein expression of PTC1 and PTC2 in DRG cells infected with lentivirus

**Figure 2:**
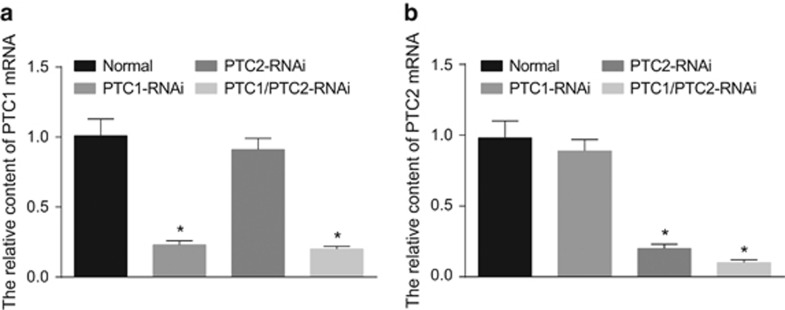
Comparisons of PTC1 and PTC2 mRNA expression in DRG cells among the normal, PTC1-RNAi, PTC2-RNAi and PTC1/PTC2-RNAi groups. Notes: (**a**) relative PTC1 mRNA expression in DRG cells among the four groups; (**b**) relative PTC2 mRNA expression in DRG cells among the four groups; PTC1, Patched 1; PTC2, Patched 2; DRG, dorsal root ganglion; *P<0.05 compared with the normal group.

**Figure 3:**
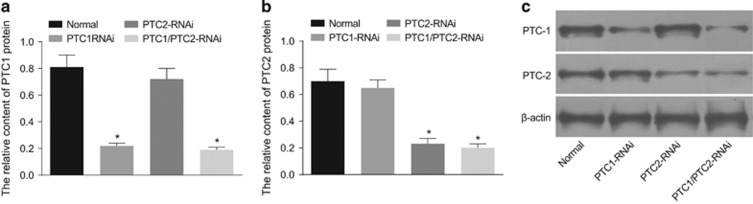
Comparisons of PTC1 and PTC2 protein expression in DRG cells among the normal, PTC1-RNAi, PTC2-RNAi and PTC1/PTC2-RNAi groups. Notes: (**a**) relative PTC1 protein expression in DRG cells among the four groups; (**b**) relative PTC2 protein expression in DRG cells among the four groups; (**c**) relative gray values of PTC1 and PTC2 proteins detected by western blotting in the four groups; PTC1, Patched 1; PTC2, Patched 2; DRG, dorsal root ganglion; RNAi, RNA interference; *P<0.05 compared with the normal group.

Two days after DRG cells were infected with RNAi lentivirus, the levels of PTC1 and PTC2 mRNA were measured, as shown in Figure 2. The PTC1-RNAi and PTC1/2-RNAi groups exhibited significantly lower PTC1 mRNA levels than the control group (both *P*<0.05). The PTC2-RNAi and PTC1/2-RNAi groups showed significantly lower mRNA levels of PTC2 than the control group (both *P*<0.05). The protein levels of PTC1 and PTC2 were determined 2 days after DRG cells were infected with lentivirus, as depicted in Figure 3. Compared with the control group, the PTC1-RNAi and PTC1/2-RNAi groups exhibited a marked decrease in PTC1 protein levels (both *P*<0.05), and the PTC2-RNAi and PTC1/2-RNAi groups displayed significantly lower PTC2 protein levels (both *P*<0.05). The above results supported successful lentivirus-mediated shRNA interference.

### Comparison of BBB scores of rats in the seven groups at different time points

Prior to SCI, all the selected rats in the study had 21 points based on the BBB scales. After SCI, all the rats exhibited paralyzed hind legs, tail dysfunctions, no responses to acupuncture, urination disorders and reduced diets (all *P*<0.05). As time progressed, the BBB scores of the SCI rats gradually increased. Seven days after injury, SCI rats could move one joint at most. Fourteen days after injury, SCI rats in the PTC1-RNAi, PTC2-RNAi and PTC1/PTC2-RNAi groups exhibited significantly increased ranges of hind limb joint motion, while those in the NC and SCI groups typically showed only mild joint motion. Twenty-one days after injury, rats in the PTC1-RNAi, PTC2-RNAi and PTC1/PTC2-RNAi groups were capable of dragging a weighted load and exhibited improved urination function. Twenty-eight days after injury, rats in the PTC1/PTC2-RNAi group placed their hind feet flat on the floor and received 9 points on the BBB scales. The recovery of rats in the PTC1/PTC2-RNAi group was significantly better than that in the NC, SCI, PTC1-RNAi and PTC2-RNAi groups (all *P*<0.05). The subsequent recovery of rats in the PTC1-RNAi and PTC2-RNAi groups was superior to that in the NC and SCI groups (all *P*<0.05). In relation to the BBB scales, rats in the NC and SCI groups had lower scores than those in the normal, sham, PTC1-RNAi, PTC2-RNAi and PTC1/PTC2-RNAi groups. No significant difference in BBB scores was observed between the NC and SCI groups (all *P*>0.05) (Table 1).

### Comparison of Shh, Gli-1, PTC1 and PTC2 mRNA expression among the seven groups

**Figure 4:**
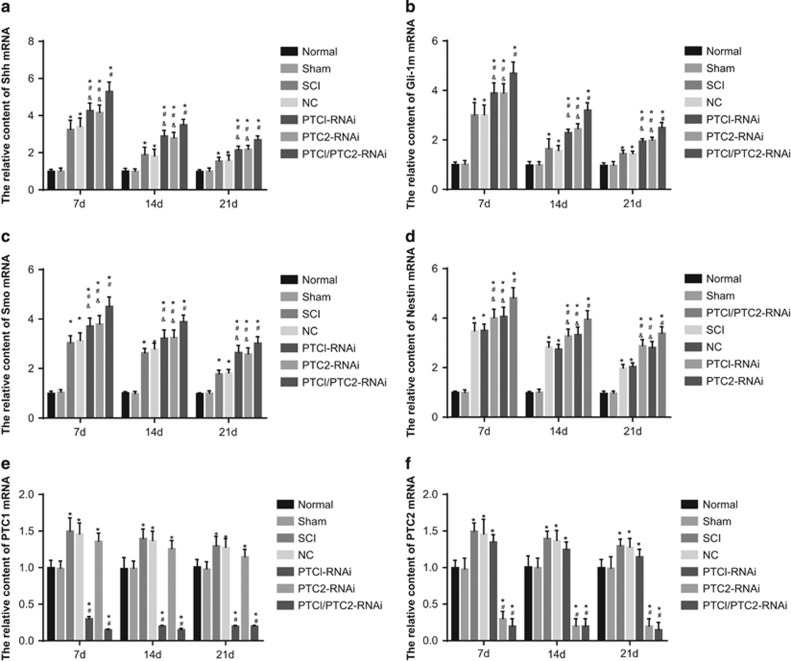
Comparison of Shh, Gli-1, PTC1, PTC2, Nestin and Smo mRNA expression among the seven groups. Notes: (**a**) relative Shh mRNA expression among the seven groups; (**b**) relative Gli-1 mRNA expression among the seven groups; (**c**) relative Smo mRNA expression among the seven groups; (**d**) relative Nestin mRNA expression among the seven groups; (**e**) relative PTC1 mRNA expression among the seven groups; (**f**) relative PTC2 mRNA expression among the seven groups; Shh, sonic hedgehog; Gli-1, glioma-associated oncogene homolog 1; PTC1, Patched 1; PTC2, Patched 2; SCI, spinal cord injury; NC, negative control; *P<0.05 compared with the normal group; ^#^*P*<0.05 compared with the SCI group; ^&^*P*<0.05 compared with the PTC1/PTC2-RNAi group.

qRT-PCR revealed evidence concerning the expression of Shh, Gli-1 and Smo in all seven groups. Rats in the SCI, NC, PTC1-RNAi, PTC2-RNAi, and PTC1/PTC2-RNAi groups showed elevated expression levels of Shh, Gli-1 and Smo, which peaked on the 7th day and remained high until the 28th day (*P*<0.05). Rats in the PTC1/PTC2-RNAi group showed the highest mRNA levels of Shh, Gli-1 and Smo among all seven groups (all *P*<0.05). In addition, the Shh, Gli-1 and Smo mRNA levels were significantly elevated in the PTC1-RNAi and PTC2-RNAi groups compared with the SCI and normal groups (*P*<0.05); however, no apparent difference was observed between the PTC1-RNAi and the PTC2-RNAi groups (all *P*>0.05). Compared with the normal group, the SCI group exhibited significantly increased Shh, Gli-1 and Smo mRNA levels (*P*<0.05). Moreover, the mRNA levels of Nestin, despite an obvious increase after SCI, were relatively elevated in the PTC1-RNAi, PTC2-RNAi and PTC1/PTC2-RNAi groups. Comparatively, the PTC1/PTC2-RNAi group exhibited the greatest increase in this regard (*P*<0.05) (Figure 4).

The PTC1-RNAi and PTC1/PTC2-RNAi groups showed significant decreases in mRNA expression of PTC1, in parallel to the normal, sham, SCI, NC and PTC2-RNAi groups (*P*<0.05). In addition, the PTC2-RNAi and PTC1/PTC2-RNAi groups also displayed rather apparent decreases in mRNA expression of PTC2 in comparison to the other five groups (*P*<0.05) (Figure 4). The SCI and NC groups exhibited the highest mRNA expression of PTC1 and PTC2, which gradually decreased as time progressed but remained significantly higher than that in the normal group (*P*<0.05).

### Comparison of Shh, Gli-1, PTC1 and PTC2 protein expression among the seven groups

**Figure 5:**
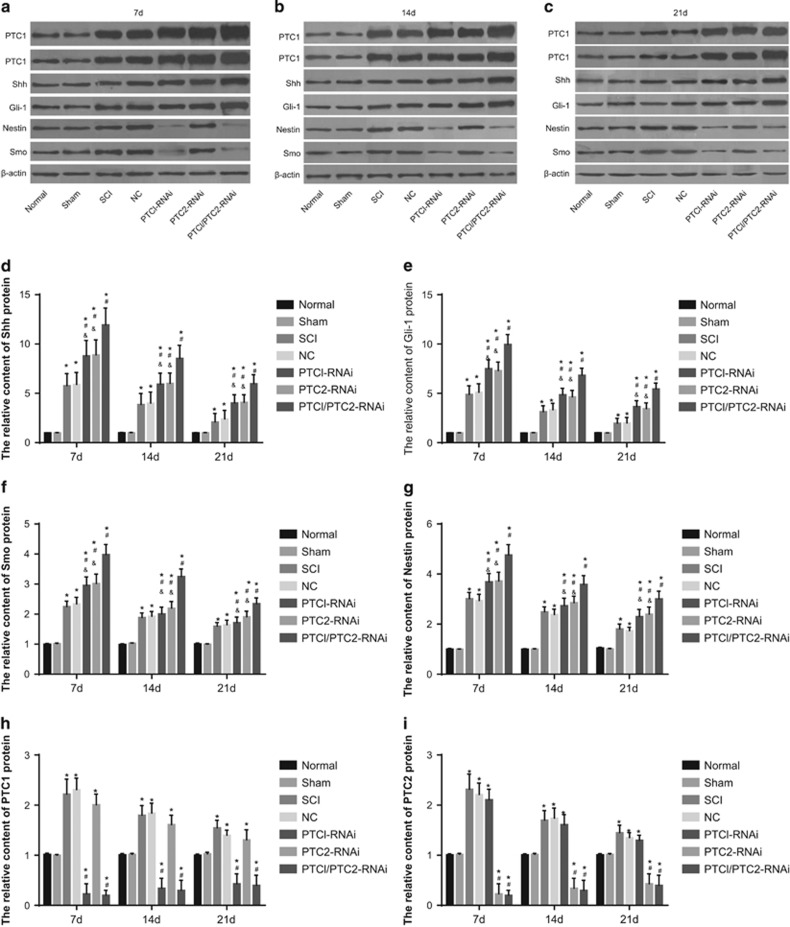
Comparison of the relative expression of PTC1, PTC2, Shh, Gli-1, Nestin and Smo proteins among the seven groups on the 7th, 14th and 28th days. Notes: (**a**) relative expression of PTC1, PTC2, Shh, Gli-1, Nestin and Smo proteins on the 7th day; (**b**) relative expression of PTC1, PTC2, Shh, Gli-1, Nestin and Smo proteins on the 14th day; (**c**) relative expression of PTC1, PTC2, Shh, Gli-1, Nestin and Smo proteins on the 21st day; (**d**) relative expression of Shh protein; (**e**) relative expression of Gli-1 protein; (**f**) relative expression of Smo protein. Relative expression of Nestin protein; (**h**) relative expression of PTC1 protein; (**i**) relative expression of PTC2 protein; Gli-1, glioma-associated oncogene homolog 1; NC, negative control; PTC1, Patched 1; PTC2, Patched 2; SCI, spinal cord injury; Shh, sonic hedgehog; *P<0.05 compared with the normal group; ^#^*P*<0.05 compared with the SCI group; ^&^*P*<0.05 compared with the PTC1/PTC2-RNAi group.

Figure 5 illustrates that the Shh, Gli-1, PTC1, PTC2, Smo and Nestin proteins were expressed in all seven groups. Following the observed increase in Shh, Gli-1, Smo and Nestin protein in the SCI rats, lower protein levels of PTC1 and PTC2 were detected. Western blotting analysis revealed the highest levels of Shh, Gli-1, Smo and Nestin protein in the PTC1/PTC2-RNAi group, in sharp contrast to the protein levels observed in the PTC1-RNAi, PTC2-RNAi, normal and SCI groups (all *P*<0.05). The PTC1-RNAi and PTC2-RNAi groups exhibited notably higher Shh, Gli-1, Smo and Nestin protein expressions than did the normal and SCI groups (all *P*<0.05). Moreover, the PTC1-RNAi and PTC1/PTC2-RNAi groups displayed a marked decrease in PTC1 protein expression levels compared with the normal, sham, SCI, NC and PTC2-RNAi groups (all *P*<0.05). Significantly decreased expression of PTC2 protein was observed in the PTC2-RNAi and PTC1/PTC2-RNAi groups compared with the normal, sham, SCI, NC and PTC1-RNAi groups (all *P*<0.05).

### Histomorphology of HE-stained spinal tissue from the seven groups

**Figure 6:**
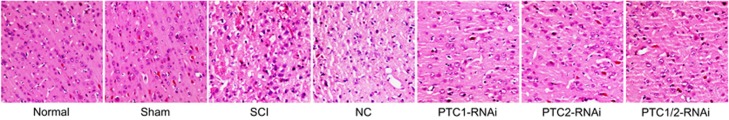
Histomorphology of HE-stained spinal tissues in the seven groups. Notes: Gli-1, glioma-associated oncogene homolog 1; HE, hematoxylin and eosin; NC, negative control; PTC1, Patched 1; PTC2, Patched 2; SCI, spinal cord injury; Shh, sonic hedgehog.

Twenty-eight days after SCI, HE staining revealed that rats in the SCI and NC groups exhibited degeneration, necrosis of neurocytes, as well as necrosis in cystic cavities. These rats also exhibited loose and disordered nerve fibers within the *substantia alba*. However, in the PTC1-RNAi, PTC2-RNAi and PTC1/PTC2-RNAi groups, the rats had less inflammation, as well as spinal cords that were regularly structured with no obvious cavities. In addition, certain areas exhibited rather noticeable nerve regeneration, as well as displays of complete structures (Figure 6).

### Comparison of NF-200-positive areas among the seven groups

**Figure 7:**
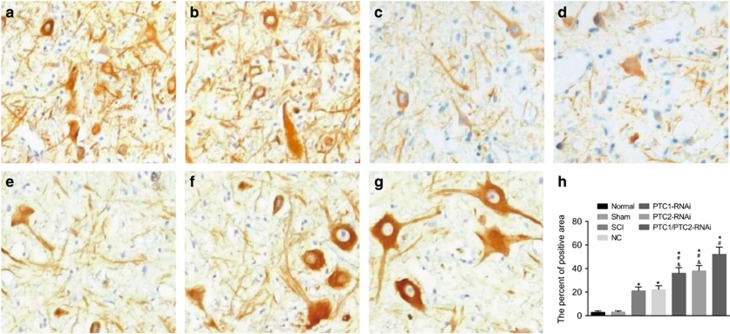
Comparison of the NF-200-positive areas detected by IHC among the seven groups (× 20). Notes: (**a**) NF-200-positive areas in the normal group; (**b**) NF-200-positive areas in the sham group; (**c**) NF-200-positive areas in the SCI group; (**d**) NF-200-positive areas in the NC group; (**e**) NF-200-positive areas in the PTC1-RNAi group; (**f**) NF-200-positive areas in PTC2-RNAi group; (**g**) NF-200-positive areas in the PTC1/PTC2-RNAi group; (**h**) comparison of the percentages of NF-200-positive areas among the seven groups detected by IHC; Gli-1, glioma-associated oncogene homolog 1; IHC, immunohistochemistry; NC, negative control; NF-200, neurofilaments-200; PTC1, Patched 1; PTC2, Patched 2; SCI, spinal cord injury; Shh, sonic hedgehog; *P<0.05 compared with the normal group; ^#^*P*<0.05 compared with the SCI group; ^&^*P*<0.05 compared with the PTC1/PTC2-RNAi group.

Twenty-eight days after SCI, immunohistochemistry in NF-200 revealed that the PTC1-RNAi, PTC2-RNAi and PTC1/PTC2-RNAi groups exhibited increased numbers of cells with relatively larger bodies accompanied by obvious nerve regeneration. Image-Pro Plus 6.0 software was used to calculate and analyze the NF-200-positive areas. The obtained results are represented in Figure 7. The PTC1/PTC2-RNAi group showed a significantly larger NF-200-positive area than the normal, sham, SCI, NC, PTC1-RNAi and PTC2-RNAi groups (all *P*<0.05). Compared with the SCI, NC and normal groups, the PTC1-RNAi and PTC2-RNAi groups also exhibited significantly larger NF-200-positive areas (all *P*<0.05). The SCI group showed a significantly larger NF-200-positive area than the normal group (*P*<0.05). Regarding the statistical analysis, there were no significant differences in this regard between the PTC1-RNAi and PTC2-RNAi groups (all *P*>0.05) (Figure 7).

### Comparison of GFAP-positive areas among the seven groups

**Figure 8:**
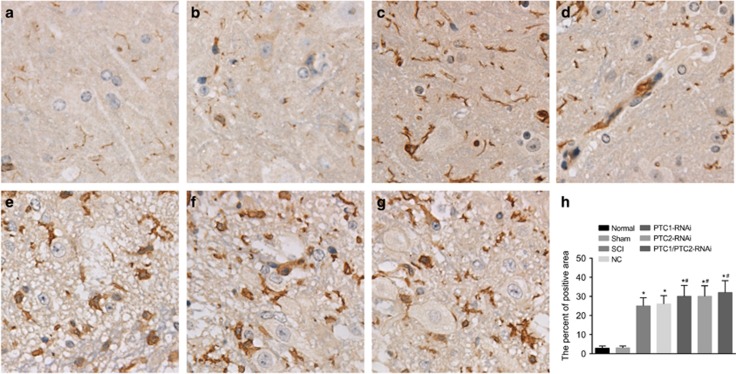
Comparison of the GFAP-positive areas detected by immunofluorescent staining among the seven groups. Notes: (**a**) GFAP-positive areas in the normal group; (**b**) GFAP-positive areas in the sham group; (**c**) GFAP-positive areas in the SCI group; (**d**) GFAP-positive areas in the NC group; (**e**) GFAP-positive areas in the PTC1-RNAi group; (**f**) GFAP-positive areas in the PTC2-RNAi group; (**g**) GFAP-positive areas in the PTC1/PTC2-RNAi group; (**h**) comparison of the percentages of GFAP-positive areas among the seven groups; GFAP, glial fibrillary acidic protein; Gli-1, glioma-associated oncogene homolog 1; NC, negative control; PTC1, Patched 1; PTC2, Patched 2; SCI, spinal cord injury; Shh, sonic hedgehog; *P<0.05 compared with the normal group; ^#^*P*<0.05 compared with the SCI and NC group.

Figure 8 shows that astrocytes in the spinal cords of the rats in all seven groups had similar expression levels of GFAP. Image-Pro Plus 6.0 software was employed to calculate and analyze the positive areas. The SCI, NC, PTC1-RNAi, PTC2-RNAi and PTC1/PTC2-RNAi groups all had significantly larger GFAP-positive areas than the normal and sham groups (all *P*<0.05). No significant differences in GFAP-positive areas were detected among the PTC1-RNAi, PTC2-RNAi, PTC1/PTC2-RNAi groups and the SCI, NC groups (all *P*>0.05).

## Discussion

Due to an increasing annual incidence, SCI remains highly prevalent worldwide.^18^ Recovery from SCI is difficult to achieve due to the inability of severed axons of the central nervous system to spontaneously regenerate.^19^ Key inhibitory obstacles that impede axonal regeneration include the glial scar and a number of myelin inhibitory molecules.^20^ Thus, there is an urgent need for progressive therapeutic strategies to promote neural regeneration.

The qRT-PCR and western blotting techniques employed demonstrated an increase in the expression of PTC1 and PTC2 after SCI. Rats in the PTC1/PTC2-RNAi group exhibited higher Shh and Gli-1 mRNA and protein expression levels. However, the rats in the aforementioned group displayed lower PTC1 and PTC2 mRNA and protein expression levels than the NC, SCI, PTC1-RNAi and PTC2-RNAi groups. This observation suggested that *PTC1* and *PTC2* gene silencing activated the Hh signaling pathway. Previous studies have indicated that the transcription of Gli1 in the nervous system and limbs is dependent on the Shh pathway.^21^ A prior study has also reported that when Gli1 is absent in nervous tissue, enteric neurons do not respond to Hh signals that can upregulate Ptch1 expression.^22^ Schnapp *et al.*^23^ reported that *PTC1* is one of the target genes of the Hh signaling pathway, which is upregulated by Shh signaling. PTC2 has been shown to modulate tumorigenesis with PTC1 haplo-insufficiency. Furthermore, medulloblastomas that develop from perturbations of PTC1 function exhibit a concomitant up-regulation of PTC2.^24^ In addition, germline inactivation of Ptch1 has been reported to be a predisposing factor in basal cell carcinoma in both humans and mice. During this study, silencing of *PTC1* and *PTC2* was accompanied by the upregulation of Shh and Gli1. This phenomenon was particularly indicative of activation of the Hh signaling pathway, which may contribute to SCI recovery. A previous report has indicated that modulating the neuro-proliferative effects of the Shh signaling cascade plays a potential role in improving the effects of CNS injury, particularly in animal models of SCI.^25^ In mature mammalian tissues, the presence of cells with progenitor function is required for regeneration after injury. The Hh pathway has been reported to expand stem cell and progenitor cell numbers in various adult tissues.^26, 27, 28^ Ochoa *et al.*^29^ reported that the activation of Hh signaling stimulates fibrogenic repair during liver injury and this activity correlates with the expansion of populations of myofibroblastic cells needed for liver cell regeneration. Moreover, studies have indicated that Shh signaling is able to induce endogenous neural precursor cells and neural stem cells, both of which are essential for neurodevelopment.^25, 30^ The data collected during the study provided sufficient evidence allowing us to deduce that the Hh pathway is an important factor in regeneration after injury. Similarly, in the present study, *PTC1* and *PTC2* gene silencing elicited a downregulation effect on the expression of PTC1 and PTC2, while upregulating Shh and Gli, thus activating the Hh signaling pathway as well as promoting neural regeneration.

The BBB scale is the most frequently used method to evaluate functional recovery in rats after SCI.^31^ Twenty-eight days after SCI, rats in the PTC1/PTC2-RNAi group achieved higher BBB scores than those in the NC, SCI, PTC1-RNAi and PTC2-RNAi groups. Furthermore, the rats in the PTC1-RNAi and PTC2-RNAi groups had higher scores than those in the SCI and NC groups. The higher BBB scores in the PTC1/PTC2-RNAi group suggested the greatest efficacy in regards to silencing *PTC1* and *PTC2*, which asserted that silencing of these genes is required to promote recovery in SCI patients. Bone marrow-derived mesenchymal stem cells (BMSCs) are a promising form of stem cell with important functions in SCI repair. It has previously been reported that Shh participates in the cartilaginous differentiation of mesenchymal cells in the spine and can promote cartilage formation in BMSCs *in vitro*.^32^ Shh can promote neuronal and BMSC survival, inhibit activation of the astrocyte lineage, facilitate axonal growth and strengthen the delivery of neurotrophic factors in BMSCs^33^ Zhang *et al.*^34^ indicated that activation of the Shh/Gli1 pathway may induce oligodendrogenesis, which could ultimately contribute to nerve recovery. Straface *et al.* have reported that the inhibition of Shh impairs the up-regulation of prototypical angiogenic agents, including stromal-derived factor (SDF)-1alpha and vascular endothelial growth factor (VEGF). This phenomenon also accounts for decreases in muscle blood flow while reducing capillary density post injury. Shh inhibition results in increased inflammation as well as poor recovery of motor function after injury.^9^ Shh is capable of inducing important transcription factors such as Gli-1, which affects Bcl-2 expression (an anti-apoptotic effector) downstream of the Shh signaling pathway.^35^ As one of the direct targets of Shh signaling, Bcl2 can be regulated by Gli-1, which when inhibited by curcumin causes reduced cell growth *in vitro* and *in vivo*.^36^ Based on the above studies, the importance of Shh and Gli during SCI recovery was subsequently deduced. Increased Shh and Gli-1 may promote nerve regeneration due to activation of the Hh signaling pathway, which ultimately contributes to SCI recovery.

In the present study, HE staining revealed clear nerve regeneration in addition to a reduction in inflammation in rats in the PTC1-RNAi, PTC2-RNAi and PTC1/PTC2-RNAi groups. In addition, rats in the PTC1-RNAi, PTC2-RNAi and PTC1/PTC2-RNAi groups exhibited increases in areas occupied by cells that were positive for NF-200 or GFAP. The Hh ligand acts as an anti-inflammatory epithelial modulator in mesenchymal inflammatory milieus, and acute modulation of Hh signaling changes inflammatory pathways in the intestinal mesenchyme.^37^ Smoothened (Smo), is a type of 7-pass transmembrane protein in the Hh pathway that is transferred to cilia upon acceptance of a ligand.^38^ Moreover, myofibroblasts are capable of secreting inflammatory mediators^39^ and are responsive to the Hh signaling pathway.^22^ The reduced expression of Hh signaling causes mislocalization of myofibroblasts during late gestation and at times in the postnatal period.^37^ It has been previously suggested that when Shh is bound to PTC1 it moves away from cilia, activating the Hh signaling pathway.^40^ Activation of the Hh signaling pathway by silencing *PTC1* and *PTC2* may reduce inflammation to promote recovery. Because NF-200 is a neural marker^41^ and both NF-200 and GFAP are expressed in differentiated neurospheres,^42^ the increase in positive areas of NF-200 and GFAP in the PTC1/PTC2-RNAi group supports the hypothesis that *PTC1* and *PTC2* gene silencing promotes nerve regeneration.

In conclusion, the present study provides significant evidence that silencing of the *PTC1* and *PTC2* genes can accelerate the recovery of rats from SCI by activating the Hh signaling pathway. These findings further suggest that silencing of *PTC1* and *PTC2* is a promising strategy for accelerating recovery in patients with SCI. However, further research is required to explore the finer mechanistic details by which SCI promotes Shh and Gli-1 expression and activates the Hh signaling pathway.

## Figures and Tables

**Table 1:** Comparison of BBB scores before and after operation among the normal, sham, SCI, NC, PTC1-RNAi, PTC2-RNAi and PTC1/PTC2-RNAi groups

*BBB scale*	*Normal group*	*Sham group*	*SCI group*	*NC group*	*PTC1-RNAi group*	*PTC2-RNAi group*	*PTCl/PTC2-RNAi group*
1 d before operation	21±0.0	21±0.0	21±0.0	21±0.0	21±0.0	21±0.0	21±0.0
1 d after operation	21±0.0	20.4±0.3	0.1±0.1*	0.1±0.1*	0.1±0.1*	0.2±0.2*	0.18±0.1*
7 d after operation	21±0.0	20.5±0.4	1.74±0.8*	1.82±0.6*	1.9±0.7*	1.9±0.6*	2.1±0.4*
14 d after operation	21±0.0	20.6±0.3	2.7±1.2*	2.8±0.7*	4.8±1.5^*,#^	4.9±1.6^*,#^	5.8±1.6^*,#^
21 d after operation	21±0.0	20.8±0.3	3.7±1.8*	3.75±1.7*	7.1±0.9^*,#^	7.5±1.8^*,#^	11.92±1.5^*,#,&^
28 d after operation	21±0.0	20.9±0.2	5.5±1.8*	5.4±1.9*	9.5±1.9^*,#^	9.8±2.1^*,#^	14.80±1.9^*,#,&^

Abbreviations: BBB, Basso, Beattie and Bresnahan; d, day; NC, negative control; PTC1, Patched 1; PTC2, Patched 2; SCI, spinal cord injury.

*P<0.05 in comparison with the normal group.

^#^*P*<0.05 in comparison with the SCI and NC groups.

^&^*P*<0.05 in comparison with the PTC1-RNAi and PTC2-RNAi groups.
